# Attitudes of laboratory animal professionals and researchers towards carbon dioxide euthanasia for rodents and perceived barriers to change

**DOI:** 10.1177/00236772211025166

**Published:** 2021-07-01

**Authors:** Michael W Brunt, Lucia Améndola, Daniel M. Weary

**Affiliations:** Animal Welfare Program, University of British Columbia, Canada

**Keywords:** Mixed methods, animal welfare, humane killing

## Abstract

Evidence indicates that carbon dioxide (CO_2_) induces negative affective states (including anxiety, fear and distress) in laboratory rodents, but many countries still accept it for euthanasia. Alternative methods (e.g. inhalant anaesthetic) may represent a refinement over CO_2_ but are not widely adopted. We conducted an online survey of Canadian and European laboratory animal professionals and researchers (*n* = 592) to assess their attitudes towards the use of CO_2_ and alternative methods for rodent euthanasia using quantitative 7-point scale (from 1 (= strongly oppose) to 7 (= strongly favour) and qualitative (open-ended text) responses. CO_2_ was identified as the most common method used to kill rodents, and attitudes towards this method were variable and on average ambivalent (mean ± SD score on our 7-point scale was 4.4 ± 1.46). Qualitative analysis revealed four themes relating to participant attitude: (a) the animal’s experience during gas exposure; (b) practical considerations for humans; (c) compromise between the animal’s experience and practical considerations; and (d) technical description of the procedure or policies. Many participants (51%) felt that there were alternatives available that could be considered an improvement over CO_2_, but perceived barriers to implementing these refinements. Qualitative analysis of these responses revealed five themes: (a) financial constraints; (b) institutional culture; (c) regulatory constraints; (d) research constraints; and (e) safety concerns. In conclusion, concerns regarding the use of CO_2_ often focused on the animal’s experience, but barriers to alternatives related to operational limitations. New research is now required on to how best to overcome these barriers.

## Attitudes des professionnels des animaux de laboratoire et des chercheurs à l’égard de l’euthanasie des rongeurs au dioxyde de carbone et obstacles au changement perçus Résumé

Les données indiquent que le dioxyde de carbone (CO_2_) induit des états affectifs négatifs (dont l'anxiété, la peur et la détresse) chez les rongeurs de laboratoire, mais de nombreux pays l'acceptent encore pour l'euthanasie. D'autres méthodes (par exemple, l'anesthésique inhalé) peuvent représenter un raffinement par rapport au CO_2_, mais ne sont pas largement adoptées. Nous avons mené une enquête en ligne auprès de professionnels et de chercheurs de laboratoires canadiens et européens (*n*= 592) afin d’évaluer leurs attitudes à l’égard de l’utilisation du CO2 et des méthodes alternatives pour l’euthanasie des rongeurs à l’aide d’_2_ et des méthodes alternatives pour l’euthanasie des rongeurs à l’aide d’une échelle quantitative de 7 points (de 1 = fortement opposé à 7 = fortement en faveur) et qualitative (texte ouvert) réponses. LE CO_2_ a été identifié comme la méthode d’euthanasie des rongeurs la plus courante, et les attitudes à l’égard de cette méthode étaient variables et en moyenne ambivalentes (le score moyen ± ET sur notre échelle de 7 points était de 4,4 ± 1,46). L'analyse qualitative a révélé quatre thèmes liés à l'attitude des participants: 1) l’expérience de l’animal pendant l’exposition au gaz, 2) les considérations pratiques pour les humains, 3) le compromis entre l’expérience de l’animal et les considérations pratiques et 4) la description technique de la procédure ou des politiques. De nombreux participants (51%) estimaient qu’il existait des solutions de rechange qui pourraient être considérées comme une amélioration par rapport au CO_2_, mais ils pensaient qu’il existait également des obstacles à la mise en œuvre de ces raffinements. L'analyse qualitative de ces réponses a révélé cinq thèmes: 1) les contraintes financières, 2) la culture institutionnelle, 3) les contraintes réglementaires 4) les contraintes de recherche et 5) les préoccupations en matière de sécurité. En conclusion, les préoccupations concernant l’utilisation du CO_2_ ont souvent porté sur l’expérience de l’animal, mais les obstacles aux alternatives étaient liées aux imitations opérationnelles. De nouvelles recherches sont maintenant nécessaires sur la meilleure façon de surmonter ces obstacles.

## Einstellungen und Ansichten von Versuchstierkundlern und -forschern bezüglich des Einsatzes von Kohlendioxid zur Tötung von Nagern und bezüglich der Hindernisse für Veränderungen Abstract

Obgleich es erwiesen ist, dass Kohlendioxid (CO_2_) bei Labornagern negative affektive Zustände (darunter Unruhe, Angst und Stress) hervorruft, gilt es in vielen Ländern jedoch nach wie vor als akzeptiertes Mittel zur Tötung. Alternative Methoden wie z. B. Inhalationsanästhesie, die im Vergleich zu CO_2_ eine Verbesserung darstellen, werden selten angewendet. Wir haben eine Online-Umfrage unter kanadischen und europäischen Versuchstierkundlern und -forschern (n=592) durchgeführt, um ihre Einstellung zur Verwendung von CO_2_ und zu alternativen Methoden zur Tötung von Nagetieren anhand einer quantitativen 7-Punkte-Skala (von 1= stark ablehnend bis 7 = stark befürwortend) und qualitativer Antworten (offener Text) zu bewerten. CO_2_ wurde als die am häufigsten verwendete Methode zur Tötung von Nagern identifiziert. Die Ansichten zu dieser Methode waren unterschiedlich und im Durchschnitt unentschieden (mittlerer ± SD-Wert auf unserer 7-Punkte-Skala war 4,4 ± 1,46). Die qualitative Analyse ergab vier Aspekte in Bezug auf die Einstellung der Teilnehmer: 1) Reaktionen/Erfahrungen des Tieres während der Gasexposition, 2) praktische Überlegungen für den Menschen, 3) Kompromiss zwischen den Reaktionen/Erfahrungen des Tieres und praktischen Überlegungen und 4) technische Beschreibung des Verfahrens oder der Richtlinien. Viele Teilnehmer (51%) waren der Meinung, dass es Alternativen gibt, die als Verbesserung gegenüber CO_2_ angesehen werden könnten, sahen aber Hindernisse bei der Umsetzung dieser Verbesserungen. Die qualitative Analyse dieser Antworten ergab fünf Aspekte: 1) finanzielle Zwänge, 2) institutionelle Kultur, 3) regulatorische Auflagen, 4) Forschungseinschränkungen und 5) Sicherheitsbedenken. Zusammenfassend lässt sich sagen, dass Bedenken bezüglich der Verwendung von CO_2_ häufig Bezug auf die Erfahrungen des Tieres nahmen, die Hemmnisse für Alternativen jedoch mit praktischen Beschränkungen zusammenhingen. Daher bedarf es weiterer Forschungsarbeit, um zu ermitteln, wie diese Hemmnisse am besten überwunden werden können.

## Actitud de los profesionales e investigadores que utilizan animales de laboratorio respecto a la eutanasia por inhalación de dióxido de carbono para roedores y obstáculos que deben superarse Resumen

Las pruebas demuestran que el dióxido de carbono (CO_2_) provoca estados emocionales negativos (como ansiedad, miedo y estrés) en roedores de laboratorio pero muchos países siguen utilizando este método para la eutanasia. Métodos alternativos (p. ej., anestesia por inhalación) pueden representar un refinamiento respecto al CO_2_ pero es una técnica todavía no generalizada. Hemos realizado una encuesta online de investigadores y profesionales canadienses y europeos que utilizan animales de laboratorio (*n*=592) para evaluar su actitud respecto al uso de CO_2_ y métodos alternativos para la eutanasia de roedores utilizando respuestas cuantitativas con una escala de 7 puntos (1= totalmente en contra, 7 = totalmente a favor) y respuestas cualitativas (texto abierto). Se identificó al CO_2_ como el método más utilizado para sacrificar roedores, y la actitud respecto a este método fue variable y ambivalente en general (la puntuación media ± de desviación estándar de nuestra escala de 7 puntos era de 4,4 ± 1,46). Los análisis cualitativos expusieron cuatro puntos relacionados con la actitud de los participantes: 1) la experiencia del animal durante la exposición al gas, 2) consideraciones prácticas para humanos, 3) compromiso entre la experiencia del animal y las consideraciones prácticas y 4) descripción técnica del procedimiento o las políticas. Muchos participantes (51%) pensaban que había alternativas disponibles que podrían considerarse como una mejora de la técnica con CO_2_ pero percibieron obstáculos para implementar estos refinamientos. Los análisis cualitativos de estas respuestas expusieron cinco puntos: 1) problemas financieros, 2) cultura institucional, 3) limitaciones en la regulación, 4) limitaciones en la investigación y 5) aspectos de seguridad. En conclusión, existen preocupaciones sobre el uso de CO_2_ que suelen centrarse en la experiencia del animal, pero hay obstáculos a la hora de implementar estas alternativas debido a las limitaciones operativas. Ahora es necesario realizar más estudios de investigación sobre cómo superar estos obstáculos.

## Introduction

Mice and rats are widely used in research; in 2017 nearly 7 million of these animals were used in the member states of The European Union^1^ and 1.5 million were used in Canada.^2^ Most of these animals were likely killed at the end of the study. It is generally agreed that laboratory animals should be killed humanely – pain, distress, fear and anxiety should be minimal or absent during the killing processes. In addition to considerations regarding the animal’s experience, preferred killing methods should have high reliability, non-reversibility, be compatible with research objectives and safe for the people performing the procedure.^3–6^ Carbon dioxide (CO_2_) is the most commonly used method to kill rats and mice; in an international meeting on laboratory animal euthanasia held on 2016, many participants reported using CO_2_ to kill rodents.^7^ This method is conditionally acceptable in Canada^3^ and the USA,^5^ and is listed as ‘appropriate’ in the Directive 2010/63/EU on the Protection of Animals Used for Scientific Purposes for the member states of The European Union.^4^

After more than 30 years of research assessing the humaneness of CO_2_ for rodent euthanasia (e.g. Blackshaw et al.^8^ and Britt^9^), its use remains controversial. For example, two recently published literature reviews arrived at contrasting conclusions; Turner and colleagues concluded that there was not enough evidence to determine whether CO_2_ killing compromises rodent welfare,^10^while Améndola and Weary concluded that CO_2_ inhalation induces negative emotions in rats likely corresponding to fear, anxiety, dyspnea, distress, and panic.^11^ Conflict also surrounds alternative methods such as inhalant anaesthetics, with some scholars arguing that that use of CO_2_ should continue,^12^ and others concluding that alternative methods are more humane.^13–15^

The lack of consensus from the research community regarding the humaneness of CO_2_ and viability of alternatives could explain why CO_2_ continues to be widely used. Implementing even well-accepted refinements to long standing practices within laboratory animal science can be a challenge. For example, more than a decade ago tunnel handling was shown to be better than tail handling as a method of physical capture for mice, reducing both aversion and anxiety induced by restraint,^16^ but tail handling of laboratory mice remains common.^17^ Additionally, animal caretakers believe the tickling of laboratory rats may benefit their welfare but implementation of the practice is low.^18^ These examples suggest that animal users in laboratories face barriers to the adoption of refinements.

The objectives of the current study were to describe (a) the attitudes of laboratory animal professionals and researchers towards CO_2_ euthanasia of rodents, and (b) the perceived barriers to implementing refinements.

## Material and methods

### Participants and recruitment

This study was approved by the University of British Columbia’s Behavioural Research Ethics Board (H19-00839). During survey development purposive theory-based sampling was employed to generate participant recruitment strategies.^19^ European and Canadian participants were targeted for recruitment because the authors had contacts within the Federation of European Laboratory Animal Science Associations, the Canadian Association for Laboratory Animal Science and the Canadian Association for Laboratory Animal Medicine. Through these contacts the survey was distributed to the respective individual members made up primarily of animal care takers, technicians, managers, veterinarians and researchers. Fifteen test participants from the University of British Columbia assessed the survey for errors and clarity. These responses were removed before launching the survey which was then active from 8 April to 22 May 2019.

Survey participants were asked about their attitudes toward the use of CO_2_ to euthanize rodents. They were asked to indicate their response, using a 7-point scale, to three similarly worded statements:

1. The use of CO_2_ to euthanize rodents is: 1, a very bad thing, 4, neither good nor bad, and 7, a very good thing.
2. The use of CO_2_ to euthanize rodents is: 1, totally appropriate, 4, neither appropriate nor inappropriate, and 7, totally inappropriate.
3. The use of CO_2_ to euthanize rodents is: 1, completely unacceptable, 4, neither acceptable nor unacceptable, and 7, completely acceptable.

In each case intermediate response options (i.e. 2, 3, 5 and 6) were indicated but not labelled. Note that for the second statement the Likert options were reversed (as a type of attention check). We excluded people who gave the same value to all three questions (e.g. 7, 7, 7), except for intermediate values (i.e. 3, 4 or 5) as in this case a consistent response could still be reasonable.

Participants were also asked about the methods they use to euthanize laboratory rodents, about perceived alternative methods, and about barriers to adopting these refinements. Qualitative responses used text boxes to record open-ended explanations of the participant’s views. Participants were also asked a series of demographic questions associated with attitudes towards animals.

We received 657 total responses. After excluding participants that failed the attention check, did not provide qualitative answers, or did not reside inside the European Union or Canada the total number of participants was 592.

### Quantitative analysis

Quantitative analyses were carried out with R (R Development Core Team, Version 3.4.1) and RStudio (RStudio, Inc., Version 1.0.136). We assessed internal consistency between the three 7-point scale statements and found high internal consistency across responses (Cronbach alpha = 0.89), so we used the mean of the three values (after reversing responses to the second question) to generate an attitude score. Lower scores reflected unfavourable views, and higher scores more favourable views. Using analysis of variance, we assessed the effects of the use of CO_2_ as primary method (yes v. no) and of participant demographics, including gender (female v. other), age (continuous in years), level of education (high school v. college/university v. graduate/professional), primary role (animal caretaker v. technician v. management v. veterinarian v. researcher v. other), sector (academic v. pharma/CRO/private v. government v. hospital/clinic v. non-profit v. other) and region (Europe v. Canada). Significant effects of factors with more than two levels were further explored using Tukey post-hoc tests. Normality of the residuals was visually assessed. Results below are reported as mean ± standard error.

### Qualitative analysis

Qualitative data were analysed by qualitative description.^20^ The authors coded a sample of participant responses from both European and Canadian participants. Codes emerged through constant comparison and axial coding and were consolidated into themes.^21^ Inter-coder reliability and codebook validity was established by one author (MB) and another researcher who independently coded a subset of data (following Guest et al.^22^). Substantial agreement was reached between the two researchers (kappa = 0.75) and consensus was reached on all remaining coded differences. Illustrative quotations were selected based on how effectively these related to the theme; participants associated with the quotes are identified in the text below using an anonymous number assigned upon entry to the survey. When quotations required editing for clarity this is indicated using square brackets around inserted words.

## Results

### Quantitative analysis

A summary of the demographic data is provided in Table 1. Of the 592 responses, 35% were from Canada and 65% from the European Union. Most responses from the European Union came from the UK (30%), Switzerland (18%), Germany (17%), Spain (7%) and France (7%). Most participants self-identified as female (67%), from the academic sector (65%) and holding a post-graduate degree (60%). Many participants reported using CO_2_ as their primary method to euthanize laboratory rodents (47%; Table 2). Additionally, most participants stated there are (52%) or may be (37%) methods which they considered improvements over CO_2_; only 12% felt that there were no such refinements available.

**Table 1.** Demographics of survey participants (*n* = 592).

Demographics		*n*	%	Attitude score (mean ± SE)
Age	18–29	57	9.6	4.21 ± 0.19
	30–39	175	29.6	4.18 ± 0.1
	40–49	174	29.4	4.56 ± 0.11
	50+	186	31.4	4.38 ± 0.11
Gender	Female	398	67.2	4.26 ± 0.07
	Male	188	31.8	4.51 ± 0.11
	Non-binary	6	1	5.94 ± 0.47
Education	High school	28	4.7	4.86 ± 0.25
	College or university	212	35.8	4.22 ± 0.1
	Masters, doctorate, DVM, MD	352	59.5	4.4 ± 0.08
Role	Animal caretaker	24	4	4.58 ± 0.26
	Technician	128	21.6	4.2 ± 0.13
	Management	114	19.3	4.43 ± 0.13
	Veterinarian	172	29.1	4.32 ± 0.14
	Researcher	121	20.4	4.45 ± 0.14
	Other	33	5.6	4.36 ± 0.29
Sector	Academic	387	65.4	4.41 ± 0.08
	Pharma, CRO, private	105	17.7	4.3 ± 0.13
	Government	36	6.1	3.82 ± 0.27
	Hospital/clinic	29	4.9	4.31 ± 0.19
	Non-profit	21	3.5	4.38 ± 0.29
	Other	14	2.4	4.83 ± 0.39
Country	Canada	209	35.3	4.21 ± 0.1
	Europe	383	64.7	4.44 ± 0.1

**Table 2.** Primary method of rodent euthanasia reported from survey of European and Canadian laboratory animal professionals and researchers (*n* = 592).

	*n*	%
CO_2_	277	46.8
Cervical dislocation	138	23.3
Isoflurane	48	8.1
Decapitation	26	4.4
Pentobarbital	20	3.4
Concussion	13	2.2
Other	27	4.6
Don't know	43	7.2
Total	592	

**Figure 1.**
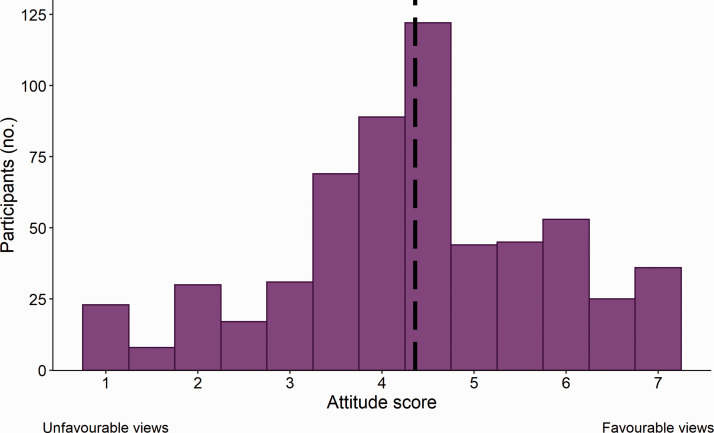
Attitude score towards the procedure of CO_2_ euthanasia of rodents from European and Canadian (*n* = 592) laboratory animal professionals and researchers.

**Figure 2.**
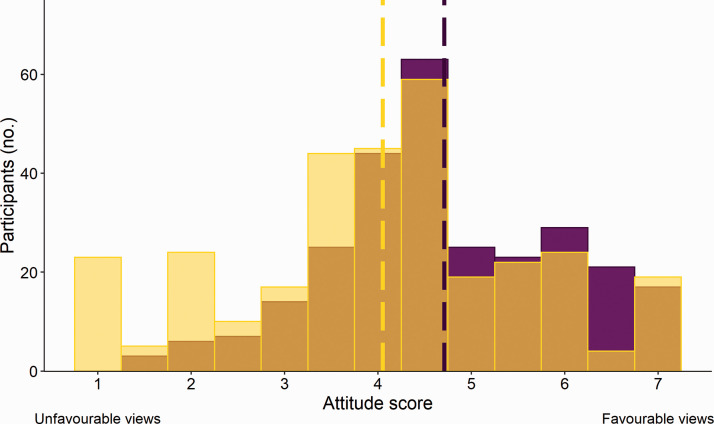
Attitude score towards the procedure of CO_2_ euthanasia of rodents from laboratory animal professionals and researchers that use CO_2_ as their primary method (purple) and that use other methods (yellow) (*n* = 592).

**Figure 3.**
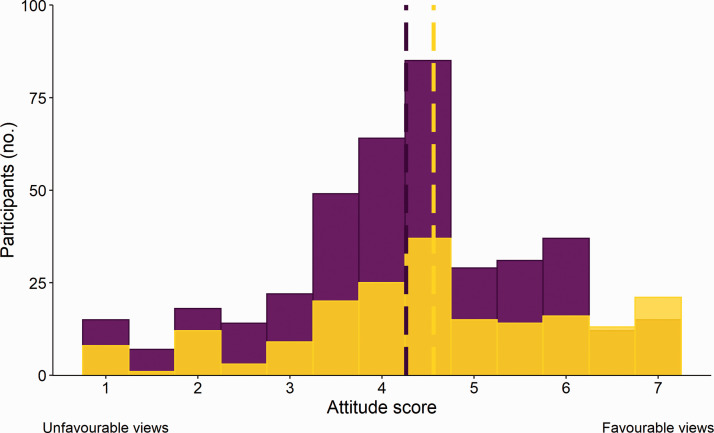
Attitude score towards the procedure of CO_2_ euthanasia of rodents from female (purple) and others (yellow) (*n* = 592) laboratory animal professionals and researchers.

The mean attitude score ranged between 1 and 7, and averaged 4.4 ± 0.06 reflecting an ambivalent attitude to CO_2_ euthanasia (Figure 1). Participants who reported using CO_2_ as their primary method showed more support for its use than those who reported using other methods (F_1, 575_ = 30.81, *p* < 0.0001; Figure 2). Female participants were slightly less supportive (F_1, 575_ = 5.13, *p* = 0.02; Figure 3) which is consistent with other studies reporting gender differences in attitudes towards animals.^23–25^ We found no effect of age (F_1, 575_ = 2.41, *p* = 0.12), education (F_2, 575_ =2.01, *p* = 0.13), role (F_5, 575_ = 0.29, *p* = 0.92), sector (F _5, 575_ = 1.46, *p* = 0.20), or region (F_1, 575_ = 1.09, *p* = 0.30).

### Qualitative analysis

Four themes emerged from the qualitative analysis of participant responses related to CO_2_ euthanasia: (a) the animal’s experience during gas exposure, (b) practical considerations for humans that used this procedure, (c) compromise between the animal’s experience and practical considerations, and (d) technical description of procedure or policies (Table 3). The majority of participants (58%) framed their response in the context of the animal’s experience. Participants framed these answers as either providing a good death (e.g. ‘*If done properly it is a humane method of euthanasia*’, participant 762) or a poor one (e.g. ‘*I feel CO_2_ is extremely stressful and painful to the animal*’, 927). One quarter of participants (25%) discussed practical considerations. For example, one participant (930) stated ‘*A quick and safe method…*’, and another respondent (480) noted that CO_2_ is ‘*Good for [euthanizing] large [numbers] of rodents*’. The practicality (e.g. ‘*It’s fast, effective, and requires minimal training*’, 259) and ease (e.g. ‘*uncomplicated method of euthanasia*’, 41) of training was also noted by participants. Some participants (12%) indicated reservations about CO_2_ euthanasia and that they saw compromise between the practical considerations and the animal’s experience. For example, participant 86 stated: ‘*I believe that CO_2_ euthanasia is associated with poor welfare; however, until more practical solutions for rodent euthanasia are available, [it] may be our best available practical option.*’ Finally, 11% of participants did not specifically offer an opinion on the procedure but instead provided a technical description. For example, participant 113 stated ‘*We still have no replacement method…*’. Others described national or institutional regulations, for example, ‘*Carbon dioxide euthanasia is a Schedule 1 method in the UK and as such is permitted*’ (87).

**Table 3.** Themes present in attitudes towards CO_2_ euthanasia of rodents from European and Canadian laboratory animal professionals and researchers (*n* = 592).

Themes	*n* ^a^	%
Animal experience	341	57.6
Practical considerations	150	25.3
Compromise	69	11.6
Technical description	63	10.6

^a^More than one theme can be present in each response.

Qualitative analysis of perceived barriers to adopting refinements identified five themes: (a) financial constraints, (b) institutional culture, (c) regulatory constraints, (d) research constraints, and (e) safety concerns (Table 4). The majority of participants (67%) described financial constraints including a lack of money, equipment, or time as a barrier to adopting refinements for rodent euthanasia. For example, participant 404 mentioned that ‘*Monetary constraints [and] infrastructure may not be suitable*’ and identified the ‘*need for [a] professionally trained operator to apply method correctly*’. The institutional culture was described as a barrier in 16.8% of responses; participants stated, for example, that ‘*We’ve always done it that way*’ (24), ‘*Resistance to change*’ (58), and ‘*People are the main barriers…*’ (42). Other responses (12%) referred to legal, regulatory and institutional policy as barriers to adopting refinements. Participant 156 identified Canada’s ‘*Controlled substance legislation*’, and participant 54 mentioned the UK’s ‘*Personal licence restrictions*’ as barriers. Some participants (11%) described research constraints, for example, stating that alternatives may disrupt scientific outcomes: ‘*Injectables may effect more organs than CO_2_ and may interfere with study results*’ (76). Finally, human or animal safety concerns were described as barriers in 10% of responses. For example, participant 205 wrote that ‘*Waste [isoflurane] gas is hazardous to humans*’.

**Table 4.** Themes present in the perceptions of barriers to the implementation of refinements for CO_2_ euthanasia of rodents from European and Canadian laboratory animal professionals and researchers (*n* = 297).

Themes	*n* ^a^	%
Financial constraints	198	66.7
Institutional culture	50	16.8
Regulatory constraints	35	11.8
Research constraints	34	11.4
Safety concerns	31	10.4

^a^More than one theme can be present in each response.

## Discussion

We found that CO_2_ continues to be a commonly used method of killing laboratory rodents, with nearly 50% of European and Canadian participants stating that this method was used most often in their facilities. Most participants had intermediate attitude scores (60% scored between 3 and 5), but those who reported using CO_2_ as their primarily methods were slightly more supportive. However, even among CO_2_ users, only 33% had favourable views (i.e. scored above 5) towards the use of CO_2_ to euthanize laboratory rodents. When asked to describe the reasons for their attitude, many participants referred to the perceived experiences of the animals. Participants varied in their interpretation of the animals’ experiences, with some believing that CO_2_ exposure was a significant source of suffering and other disagreeing. Thus, it is reasonable to infer that many participants who use CO_2_ as a primary euthanasia method are not convinced that this method provides a good death. These results are consistent with the ongoing debate within the scientific community where some authors argue that CO_2_ euthanasia causes suffering in rodents^11^ and others argue there is insufficient evidence to draw conclusions regarding the method.^10^ The current study did not assess if the information used by participants to interpret the animals’ experiences came from their knowledge of the scientific literature, personal experience, or information provided by regulatory bodies. Future studies should consider what and how information is used by laboratory animal professionals to inform their views.

Some participants explained their attitude towards CO_2_ killing in relation to practicality of the method and others weighed the animal experiences against practical considerations. These participants seemed to value the animal’s experience but often prioritized the practicality of training or existing availability. Previous research has shown a bias towards practicality when participants are faced with uncertainty,^26^ efforts to increase consensus among professionals may attenuate participant uncertainty and bias.

We are unsure of how to interpret responses from participants who simply offered technical descriptions when justifying their attitudes. This might reflect a lack of understanding of our questions, or perhaps more substantially an uneasiness with introspection on this topic. Research has shown that survey participants often use little cognitive effort in their responses and instead rely on heuristics;^27^ it is easier to substitute the substantive question (‘what should I do’) with the more simple question (‘what am I allowed to do’) when asked to justify morally relevant actions. Other methodologies that allow for probing of participant responses may provide a better understanding of the views of these participants.

Many participants believed that refined killing procedures exist, but barriers prevented their implementation. Interestingly, perceived financial constraints were identified by two-thirds of participants, and another 10% identified regulatory constraints as a barrier to adopting these refinements. This result is of special interest as both Canadian^28^ and European Union^4^ regulations specifically require committees approving animal use to implement welfare refinements. Institutions that conduct animal research may be reluctant to financially support refinements when the implementation of current practices meets regulatory obligations. Our study highlights the need for additional research to specifically explore the role financial resources plays in the implementation of animal welfare refinements.

Interference with research paradigms, animal or human safety concerns, and the culture within institutions were all identified by participants as barriers to change. In some cases, refined rodent euthanasia procedures that compromise research data or endanger the health and wellbeing of humans or animals cannot be implemented. However, in these cases efforts should continue to investigate risk mitigation strategies to enable deployment of refinements. Similar to previous research, the current study has identified organizational reasons for resistance to change.^29^ Future research should specifically focus on the cultural aspects of laboratory environments that create barriers to change.

A lack of scientific evidence did not emerge as a theme in responses regarding barriers to adopting refinements; this result suggests that new scientific research addressing the humaneness of CO_2_ killing is unlikely to help laboratory animal professionals. Interestingly, participants in a recent survey that explored the implementation of handling techniques in laboratory mice did identify a lack of scientific research as a barrier.^17^ It is possible that more in depth qualitative research methods would identify specific gaps in the science that require new research in euthanasia, but the results of the current study suggest that the focus for new research should be on understanding and addressing barriers to adopting refinements.

The current survey had several limitations. We recruited participants through email distribution; we recognize the potential for sampling bias, as participants with a particular interest in the topic might have been more willing to participate in the survey. In this case we might have expected to see many responses who were either strongly supportive or strongly opposed to the method. In contrast, we found that most participants were ambivalent, and their detailed qualitative responses indicated that they often held nuanced views understanding both the welfare impact of the procedure and practical constraints. Our participants were predominantly older, female, educated and working in an academic environment; we did not have access to the demographic breakdown of the populations from which we recruited. We encourage future studies to obtain a better estimate of population characteristics (for example, using the records for the professional associations whose memberships we recruited from). Our study population was based in Europe and Canada, limiting the ability to generalize results to other regions of the world; further research should include participants from other counties that are major users of research animals, including the USA and China.

## Supplemental Material

sj-xlsx-1-lan-10.1177_00236772211025166 - Supplemental material for Attitudes of laboratory animal professionals and researchers towards carbon dioxide euthanasia for rodents and perceived barriers to change Click here for additional data file. Supplemental material, sj-xlsx-1-lan-10.1177_00236772211025166 for Attitudes of laboratory animal professionals and researchers towards carbon dioxide euthanasia for rodents and perceived barriers to change by Michael W Brunt, Lucia Améndola and Daniel M. Weary in Laboratory Animals

sj-pdf-2-lan-10.1177_00236772211025166 - Supplemental material for Attitudes of laboratory animal professionals and researchers towards carbon dioxide euthanasia for rodents and perceived barriers to change Click here for additional data file. Supplemental material, sj-pdf-2-lan-10.1177_00236772211025166 for Attitudes of laboratory animal professionals and researchers towards carbon dioxide euthanasia for rodents and perceived barriers to change by Michael W Brunt, Lucia Améndola and Daniel M. Weary in Laboratory Animals
