# Supplementary material for: Attitudes of laboratory animal professionals and researchers towards carbon dioxide euthanasia for rodents and perceived barriers to change
Source: Lab Anim. 2021 Jul 1;55(6):531–9. doi: 10.1177/00236772211025166 (PMC8669199; doi:10.1177/00236772211025166)
Supplement: sj-pdf-2-lan-10.1177_00236772211025166 - Supplemental material for Attitudes of laboratory animal professionals and researchers towards carbon dioxide euthanasia for rodents and perceived barriers to change [file sj-pdf-2-lan-10.1177_00236772211025166.pdf]

Attitudes of laboratory animal professionals and researchers towards carbon dioxide euthanasia for rodents and perceived barriers to change

Supplementary Materials

Supplementary Table 1. Primary data

[insert S2 Primary Data.xlsx]
